# The β-glucosidase secreted by *Talaromyces amestolkiae* under carbon starvation: a versatile catalyst for biofuel production from plant and algal biomass

**DOI:** 10.1186/s13068-018-1125-9

**Published:** 2018-04-27

**Authors:** Juan Antonio Méndez-Líter, Laura Isabel de Eugenio, Alicia Prieto, María Jesús Martínez

**Affiliations:** Department of Microbial and Plant Biotechnology, Centro de Investigaciones Biológicas, CSIC, Ramiro de Maeztu 9, 28040 Madrid, Spain

**Keywords:** Fungi, Glycosyl hydrolases, Saccharification, Laminarinase, Biofuels, Carbon starvation

## Abstract

**Background:**

In the last years, the most outstanding trend for obtaining high added-value components and second-generation (2G) biofuels consisted on exploitation of plant biomass. But recently, 3G biofuels, based in algae biomass, have emerged as a great alternative for production of energy.

**Results:**

In this work, a versatile β-glucosidase from the ascomycete fungus *Talaromyces amestolkiae* has been purified, characterized, and heterologously expressed. The synthesis of this β-glucosidase (BGL-3) was not induced by cellulose, and the presence of a specific carbon source is not required for its production, which is uncommon for β-glucosidases. BGL-3, which was obtained from a basal medium with glucose as carbon source, was profusely secreted under carbon starvation conditions, which was corroborated by qRT-PCR assays. BGL-3 was purified from *T. amestolkiae* cultures in one step, and biochemically characterized. The enzyme showed high thermal stability, and very high efficiency on *p*NPG (*K*_m_ of 0.14 mM and *V*_max_ of 381.1 U/mg), cellobiose (*K*_m_ of 0.48 mM and *V*_max_ of 447.1 U/mg), and other cello-oligosaccharides. Surprisingly, it also showed remarkable ability to hydrolyze laminarin, a β-1,3-glucan present in algae. The recombinant enzyme, obtained in the yeast *Pichia pastoris,* exhibited kinetic and physicochemical properties similar to those found for the native protein. Enzyme efficiency was examined in wheat straw saccharification processes, in which BGL-3 worked better supplementing Celluclast 1.5L than the commercial cellulase cocktail N-50010. Besides, BGL-3 hydrolyzed laminarin more efficiently than a commercial laminarinase.

**Conclusions:**

A very efficient 1,4-β-glucosidase, which also showed activity over 1,3-β-glucose bonds, has been produced, purified, and characterized. This is the first report of such versatility in a 1,4-β-glucosidase. The application of this enzyme for saccharification of wheat straw and laminarin and its comparison with commercial enzymes suggest that it could be an interesting tool for the production of 2G and 3G biofuels.

**Electronic supplementary material:**

The online version of this article (10.1186/s13068-018-1125-9) contains supplementary material, which is available to authorized users.

## Background

The increase in world population, the expanding economy, and the non-renewable nature of fossil fuels are among the main reasons threatening the supply of energy to cover our needs. Industrial production of biofuels is a promising alternative to reduce our current dependence on petroleum [1].

In this context, cellulose is the most abundant potential source of renewable energy on earth. Its transformation into glucose is considered the key step in the production of biofuels from lignocellulosic biomass, and it determines the performance of the rest of the process. This transformation requires the synergistic action of three enzyme classes, endoglucanases, cellobiohydrolases, and β-glucosidases (BGLs), to hydrolyze the β-1,4 bonds of cellulose. Biological conversion of cellulosic biomass is a green way to produce second-generation (2G) ethanol and other chemicals, and it does not compete with human food resources used for first-generation bioethanol production, thus ending the controversial issues of food-versus-fuel and use of arable lands [2].

Recently, 3G biofuels, from algae biomass, have received considerable attention [3] for their advantages over traditional production pathways for biofuels: (i) algae do not need agricultural lands, since they can grow in swamp areas; (ii) they do not require fresh water, an important factor taking into account that fresh water is limited. Moreover, algae are not a seasonal crop like corn, since they can be cultured all year, making it a more sustainable energy source than first- and second-generation biofuels. Algae could be applied to obtain a wide spectrum of products such as biodiesel, bioethanol, biohydrogen, and biomethane [4]. Bioethanol production from algae has a notable potential due to the absence of lignin and hemicellulose, as compared to lignocellulosic plants, which could favor the degradation of polysaccharides to get glucose for fermentation processes. Numerous species have been studied for this objective. For example, green algae including *Spirogyra* sp. and *Chlorococcum* sp. accumulate high levels of polysaccharides like starch, which could be fermented to bioethanol [5]. One of the most promising species is represented by brown algae, of the genus *Laminaria*, for containing high quantities of laminarin. This polysaccharide was first discovered in *Laminaria digitata* and it later showed to be the main food reserve of this type of algae, being very abundant in their fronds. Structurally, laminarin is a predominantly linear β-(1,3)-glucan that can contain few branches of mannitol or glucose attached at *O*-6 positions of the main chain, and then it is a potentially nice candidate to produce bioethanol 3G [6]. With this perspective, enzymes used in 2G bioethanol processes, like cellulase cocktails, including β-glucosidases, could represent a good alternative for efficient liberation of glucose from algae polysaccharides.

Traditionally, relatively pure forms of commercial cellulose and pretreated plant biomass have been used as carbon sources for the induction of cellulolytic enzymes [7]. However, although these have been reported as the best ways to produce this type of enzymes, the substrates are usually expensive. Thus, the discovery of organisms that secrete robust and efficient cellulases without these requirements could be an interesting progress for cheapening the enzyme cost.

Fungal species from *Penicillium* and *Talaromyces*, their perfect state, are well known for producing a wide variety of β-glucosidases, with good characteristics, such as thermostability, glucose tolerance, and high efficiency towards diverse substrates [8–11]. Some recent reports deal with *T. amestolkiae,* that secretes high levels of cellulases growing in media with different carbon sources [12], and a β-glucosidase (BGL2) with a cellulose binding domain from this fungus, has proved to efficiently degrade cellulose from brewers’ spent grain [13]. This work presents the production, characterization, and catalytic versatility of a novel β-glucosidase from this fungus and its role for bioethanol 2G and 3G production.

## Results and discussion

### BGL-3 production and purification

The potential of *T. amestolkiae* as a β-glucosidase producer has been recently reported, revealing that it secreted at least two different β-glucosidases [12]. One of them was induced exclusively by cellulosic substrates (g3821), while the other one (g377) was produced in the four carbon sources tested (Avicel, glucose, xylan, or pretreated wheat straw). This phenomenon is peculiar since, usually, cellulolytic enzymes require cellulose or its derivatives to be induced, and they are repressed by glucose or other easily metabolizable carbon sources [14].

In the current work, the β-glucosidase activity increased drastically when the carbon source (glucose) was consumed, reaching its maximal level at 7 days of incubation (1.8 U/mL). The crude enzyme, obtained from the culture medium after ultrafiltration and dialysis, was loaded in a HiTrap Capto adhere cartridge and three peaks with β-glucosidase activity were detected. During the chromatographic run, a small peak that eluted during the first part of the NaCl gradient (13% of the total BGL activity) was separated from a big one that eluted with 100% NaCl and contained the bulk of the retained proteins, but only around 4% of the BGL activity. However, most of the activity (around 83%) eluted during column re-equilibration with the buffer.

**Fig. 1:**
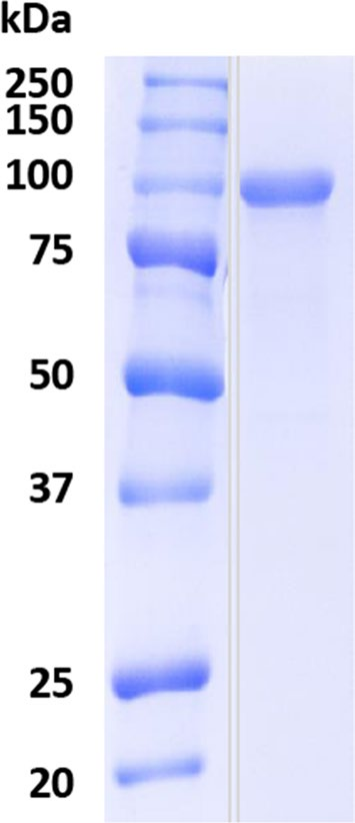
SDS-PAGE of BGL-3 from *T. amestolkiae* purified from Mandels medium using glucose as carbon source

Sodium dodecyl sulfate–polyacrylamide gel electrophoresis (SDS-PAGE) analysis of the peak with the highest β-glucosidase activity showed a single protein band of around 100 kDa (Fig. 1), demonstrating that a protein, named as BGL-3, was purified to homogeneity in a single purification step. This fact could be explained because the cartridge used for separation contains a multimodal anion exchange separation bed, combining the properties of traditional anion exchangers with the different types of intermolecular forces related with the hydrophobicity of BGL-3. The final purification yield after concentration, dialysis, and purification, was 41% (Table 1). Peptide mass fingerprint analysis of BGL-3 disclosed that this enzyme was β-glucosidase g377, the major protein produced in cultures with glucose as carbon source.

**Table 1:** Purification of the BGL-3 secreted in Mandels medium with glucose as carbon source by *T. amestolkiae* cultures

BGL-3 purification				
Step	Total protein (mg)	Total activity (U)	Specific activity (U/mg)	Yield (%)
Crude extracts	1.2	25.9	21.5	100
HiTrap Capto adhere	0.7	10.8	42.4	41.7

### Physicochemical properties of BGL-3

The molecular mass of the BGL-3 monomer, determined by MALDI-TOF, was 107.0 kDa, which coincided fairly well with the SDS-PAGE data (approximately 100 kDa). As these techniques cause dissociation of non-covalent aggregates, the native protein was analyzed by size exclusion chromatography. In these conditions, a molecular mass around 222 kDa, was calculated, suggesting that BGL-3 is a non-covalent, functional dimer. The molecular mass expected from the amino acid sequence of BGL-3 (89.5 kDa) was lower than the empirical value observed for the monomer of this protein, which can be due to protein glycosylation. This modification has already been reported for other extracellular glycosyl hydrolases from this fungus [15]. Subtracting the theoretical mass value from that determined by MALDI-TOF, an approximate glycosyl content of 16% can be deduced.

The pI value determined by isoelectric focusing (7.4) and that theoretically predicted (4.8) are different, and glycosylation can be partially responsible of this fact [16], as the glycosyl chains can produce a shielding of surface charges. However, this difference in pI could also be due to other reasons like the uneven distribution of charged amino acids in the protein, with more negative charges being buried in the protein core.

**Fig. 2:**
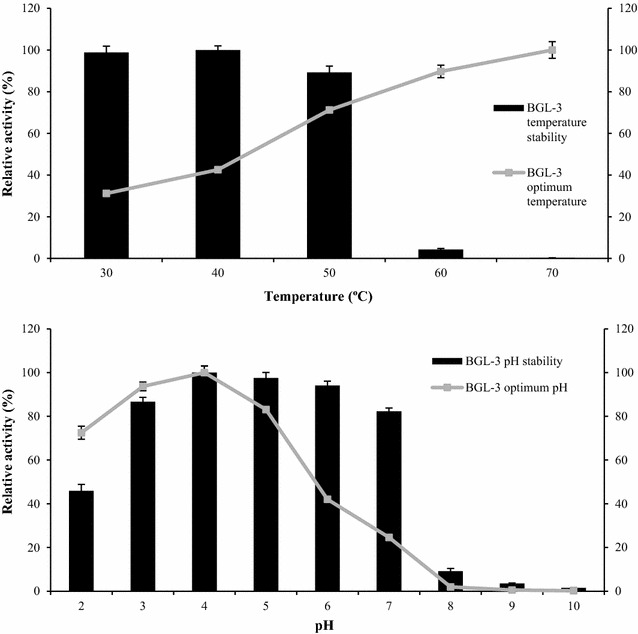
Stability and optimal pH and temperature of BGL-3, using 0.1% pNPG as substrate. Lines indicate the optimum temperature and pH values for enzyme activity; bars show its stability after 72 h in a range of temperatures from 30 to 70 °C or in a range of pH from 2 to 10. For temperature stability assays, the enzyme was incubated with sodium acetate buffer pH 4.0, 100 mM. For pH stability tests, the enzyme was incubated at the appropriate pH in Britton–Robinson buffer, 100 mM at 4 °C. All reactions were performed in triplicate

The effect of temperature and pH on BGL-3 activity were also evaluated (Fig. 2). Optimum activity of BGL-3 was obtained at pH 4 and 70 °C. Higher temperatures and basic pHs produced a fast inactivation of the enzyme. Protein stability was measured for a 72-h period, observing that BGL-3 was active in a pH range from 2 to 7, and between 30 and 50 °C, losing activity quickly at higher temperatures. Temperature and pH are essential factors for enzymes and establishing their optimum conditions is very important to evaluate a potential industrial application. Many studies have been conducted on the search of β-glucosidases with enhanced properties, such as thermostability [17–19]. Among the biochemical characteristics evaluated for BGL-3, its main advantage is its good thermal tolerance, since this enzyme was fully active at 50 °C for 72 h.

### Substrate specificity

In hydrolysis reactions, BGL-3 showed very high efficiency and good affinity on *p*-nitrophenyl-α-d-glucopyranoside (*p*NPG), *o*-nitrophenyl-α-d-glucopyranoside (*o*NPG), and cello-oligosaccharides from cellobiose to cellohexaose (Table 2). It is also remarkable that it was very active towards substrates without β-1,4 linkages, like laminaribiose and gentiobiose. However, only a residual activity was observed towards 4-nitrophenyl-β-d-xylopyranoside, maltose, and 4-nitrophenyl-α-d-glucopyranoside and no activity was detected on 4-nitrophenyl-β-d-galactopyranoside, 4-nitrophenyl-α-d-galactopyranoside, 4-nitrophenyl-α-L-rhamnopyranoside, 4-nitrophenyl-β-d-fucopyranoside, lactose, or sucrose. The BGL-3 activity was also assayed on polysaccharides: Avicel, carboxymethyl cellulose (CMC), beechwood xylan, and laminarin (Additional file 1: Table S1). It showed a low but remarkable activity on Avicel, CMC, and xylan and, unexpectedly, the enzyme had high activity against laminarin (Table 2). This β-(1,3)-glucan is specifically hydrolyzed by laminarinases (EC 3.2.1.6). To further study this interesting feature, the conditions for BGL-3 crystallization are currently being set. However, a preliminary 3D model of BGL-3 based on a β-glucosidase from *Aspergillus aculeatus* was constructed and compared to that of a barley β-d-glucan glucohydrolase isoenzyme in complex with 4′-nitrophenyl 3I-thiolaminaritrioside (SMTL ID 1j8v.1) (Additional file 1: Figure S2). Alignment of both models disclosed that the active site from BGL-3 matched the amino acids involved in substrate binding in the laminarinase. This preliminary analysis suggests that one of the possible explanations to the laminarinase activity of BGL3 is the structural similarity between its active site and that of some strict laminarinases, but further experiments are required and will be developed in order to determine it.

**Table 2:** Kinetic parameters of BGL-3 from *T. amestolkiae* against different substrates. All reactions were performed in triplicate

Substrate	*K*_m_ (mM)	*V*_max_ (U/mg)	*k*_cat_ (s^−1^)	*k*_cat_/*K*_m_ (mM^−1^ s^−1^)
*p*NPG	0.14 ± 0.1	381.1 ± 4.1	1359.4	9710.2
*o*NPG	0.14 ± 0.1	142.1 ± 3.6	506.9	3425.2
Cellobiose	0.48 ± 0.1	447.1 ± 7.0	1594.6	3308.4
Cellotriose	0.80 ± 0.1	275.7 ± 4.2	983.3	1216.9
Cellotetraose	0.35 ± 0.1	373.0 ± 5.0	1330.4	3779.5
Cellopentaose	0.32 ± 0.1	408.6 ± 6.9	1457.2	4442.7
Cellohexaose	0.57 ± 0.1	374.1 ± 3.0	1334.2	2304.4
Laminaribiose	6.34 ± 0.2	290.8 ± 5.3	1037.1	163.4
Gentiobiose	9.77 ± 0.3	299.2 ± 3.8	1067.2	109.1
Laminarin from *L. digitata*	1.21 ± 0.1	142.4 ± 1.0	253.9	211.5
Laminarin from *L. hyperborea*	1.1 ± 0.2	139.0 ± 2.0	247.9	225.3

β-glucosidases have traditionally been divided into three groups: cellobiases, which have high substrate specificity towards cellobiose, aryl-β-glucosidases, with very high specificity towards synthetic substrates as *p*NPG, and β-glucosidases with broad substrate specificity, acting on these two types of substrates and other oligosaccharides. Most β-glucosidases are placed in this last category, but it is remarkable that most of these enzymes are quite more active on *p*NPG than on cellobiose and other cello-oligosaccharides, which are their natural substrates [7]. For production of 2G bioethanol, where their main substrates are cellulosic oligosaccharides, the search for efficient cellobiases is still a challenge. To our knowledge, the BGL-3 from *T. amestolkiae* is the most efficient BGL against cello-oligosaccharides of three or more glucose units, and only rBgl4 from *Penicillium funiculosum* has similar efficiency for degradation of cellobiose (Table 3).

**Table 3:** Comparison of the kinetic parameters of *T. amestolkiae* BGL-3 with those reported for other fungal β-glucosidases, using cellobiose as substrate. All reactions were performed in triplicate

Organism	Enzyme	*K*_m_	*k*_cat_	*k*_cat_/*K*_m_	References
*Penicillium purpurogenum*		5.1	1395^a^	273^a^	[8]
*Talaromyces leycettanus*	BGL3A	10.4	786	75	[11]
*Aspergillus fumigatus*	rBgl3	2.2	114	52	[32]
*Penicillium funiculosum*	rBgl4	1.2	4513^a^	3610^a^	[10]
*Myceliophthora thermophila*	MtBgl3a	2.6	46^a^	17^a^	[18]
*Aspergillus oryzae*	HGT-BG	7.0	252	36	[34]
*T. amestolkiae*	BGL-3	0.5	1594	3308	This work

^a^ Calculated from data provided in the original article

### DNA and amino acid sequence of BGL-3

*T. amestolkiae* genome [12] was searched to deduce the sequence of BGL-3, which was classified as a β-glucosidase from the GH3 family. Introns and exons identification was performed by comparison with transcriptomic data from similar sequences using BlastN. According to these data, the *bgl3* gene contains three introns, with a sequence of 2571 bp, coding for a protein of 857 amino acids (Additional file 1: Figure S1).

**Fig. 3:**
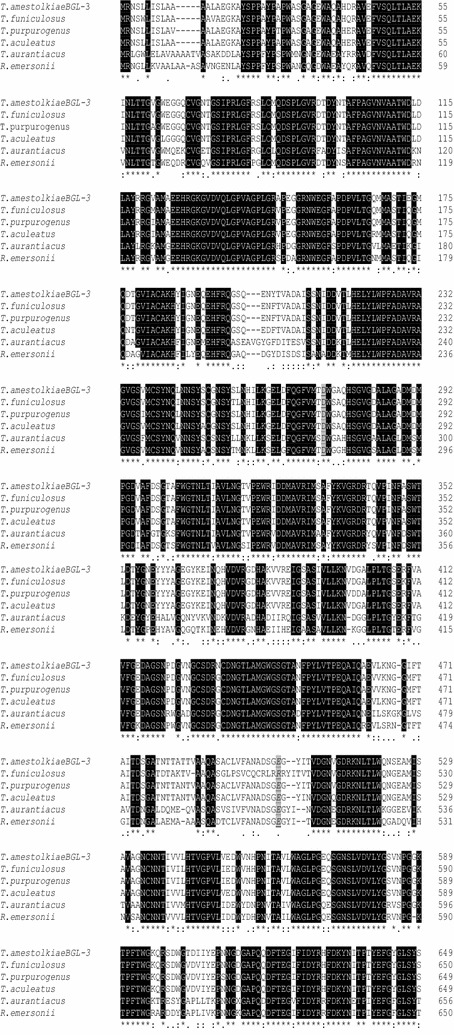
Protein sequence alignment of BGL-3 and other GH3 β-glucosidases, using Clustal Omega. The black-highlighted residues indicate fully conserved regions. The catalytic residues are indicated in gray

A homology search with BLASTP revealed 94, 95, 95, 69, and 74% amino acid identity of BGL-3 with the BGLs of *Talaromyces funiculosus* (AFU91382.1), *Talaromyces purpurogenus* (ACV87737.1), *Talaromyces aculeatus* (AGA96121.1), *Thermoascus aurantiacus* (ABX79553.1), and *Rasamsonia emersonii* (XP_013330184.1), respectively. These data suggest that BGL-3-like proteins are well conserved among ascomycetes (Fig. 3).

### Expression of *bgl*-*3* gene under carbon starvation

In a previous work, we demonstrated that the fungus secreted detectable amounts of BGL when glucose was used as carbon source, although this activity appeared upon glucose depletion (after 24 h of incubation). Besides, we detected repression of BGL activity when glucose pulses were added [12]. These results suggested that *T. amestolkiae* also released some β-glucosidase activity in the absence of cellulose. To confirm this, BGL activity and biomass were monitored at very short times in cultures with glucose (Additional file 1: Figure S3). The fungus reached its maximum growth after 12–24 h. BGL activity was detected in very low amounts during the first hours of incubation, and increased drastically once the culture reached the stationary phase.

**Fig. 4:**
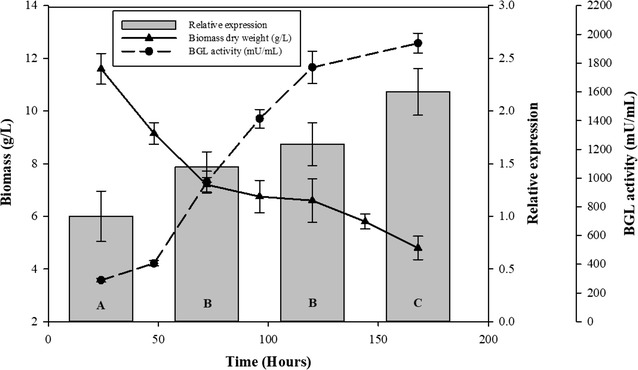
Comparison of qRT-PCR analysis of *bgl*-*3* expression and biomass over the time in cultures with Mandels medium supplemented with glucose. Samples were taken periodically to determine β-glucosidase activity against 0.1% *p*NPG, relative expression by qRT-PCR, and biomass dry weight. Data represent the mean of six replicates. Statistically significant differences between means were determined by the Tukey HSD test. Different letters in qRT-PCR results (A, B, C, and D) indicate significant differences at a *P* value of 0.05

To further study how BGL-3 is synthesized during a prolonged carbon starvation period, a transcriptomic analysis was performed. The biomass of *T. amestolkiae* was monitored in the culture over 7 days of incubation. The residual biomass determined in 7-day-old cultures was around 30% of the initial amount. A qRT-PCR analysis of *bgl3* indicated that its expression increases over time (Fig. 4), reaching a maximum transcription level on day 7, which coincided in time with the highest level of extracellular BGL activity. This confirms the accumulation of BGL-3 on carbon-deficient cultures. It is interesting to note that *bgl3* expression grows 2.1-fold from day 1 to day 7, showing that the fungus increased the synthesis of this protein when the scarcity of carbon source persists over time. Few studies have been performed to investigate the effects of carbon starvation on fungal cultures. White et al. [20] remarked the presence of extracellular hydrolase activity in these conditions. The induction of hydrolases, including glycosidases, has been proposed as a key event in the aging of fungal cultures during carbon shortage. The most detailed works developed with fungi have been conducted with *Aspergillus niger*. The studies of Nitsche et al. [21], and Van Munster et al. [22], revealed the ability of filamentous fungi to produce enzymes when the carbon source was consumed. The transcriptomic analysis of carbon-starved cultures of *A. niger* confirmed the expression of genes encoding CAZymes, including those responsible for the presence of BGL activity, which agrees with the production of BGL-3 by *T. amestolkiae* under carbon deprivation.

### Heterologous expression of BGL-3 in *P. pastoris*

**Fig. 5:**
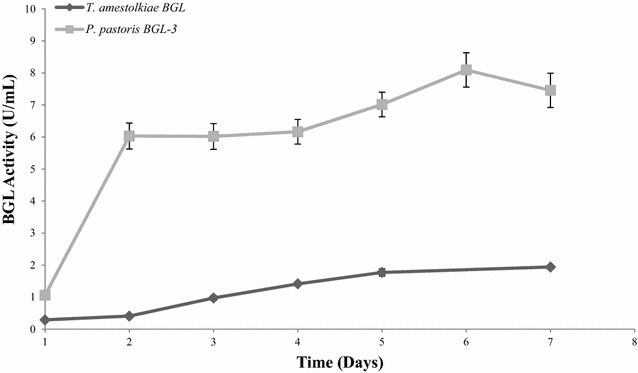
β-glucosidase production by *T. amestolkiae* or *P. pastoris*. Samples were taken daily and activity was measured against 0.1% (w/v) *p*-nitrophenyl-β-d-glucopyranoside (*p*NPG, Sigma) in sodium acetate buffer 100 mM, pH 4.0, at 70 °C. All reactions were performed in triplicate

Due to the interesting properties of BGL-3 in terms of kinetic constants and physicochemical properties, the protein was expressed in the yeast *P. pastoris* with the aim of improving its production levels. After *P. pastoris* transformation, several positive clones were screened for β-glucosidase activity, selecting clones 34 and 20 as the highest producers in liquid cultures. Maximal β-glucosidase activity (8.1 and 7.9 U/mL, respectively) was found in 7-day-old YEPS cultures. These values are 4.5-fold higher than the total β-glucosidase activity detected in cultures of *T. amestolkiae* in Mandels medium with glucose (Fig. 5). The concentration of BGL-3 in *P. pastoris* supernatants was 21.2 mg/L for the clone with the highest production. The first trial to purify the recombinant BGL-3 (BGL-3*) was done using the same protocol applied for purification of the native enzyme. However, BGL-3* was abnormally distributed throughout all fractions recovered, and it was necessary to change the procedure. The chromatographic separation on this cartridge relies on the combination of anionic exchange and hydrophobic interactions, and thus the different glycosylation patterns of the native and the recombinant enzyme can be responsible for their different retentions in this bimodal column. BGL-3* was completely purified in two steps: anion-exchange chromatography on a HiTrap QFF cartridge, followed by size exclusion chromatography. In spite of this, the overall yield for production and purification of the recombinant enzyme was 7.5-fold higher than for the native protein (54% of initial activity, Additional file 1: Table S2). As revealed by SDS-PAGE, BGL-3* had a molecular mass slightly superior as that of the native form, probably due to increased glycosylation in the yeast.

The biochemical and kinetic properties of the recombinant protein were virtually the same as those of the native BGL-3 in terms of pH, temperature, and substrate specificity. We checked with special interest its activity on laminarin, concluding that the kinetic constants of BGL-3* on this β-1,3 glucan were very similar to those of the native enzyme, which confirms their catalytic versatility.

### BGL-3 for wheat straw saccharification

**Fig. 6:**
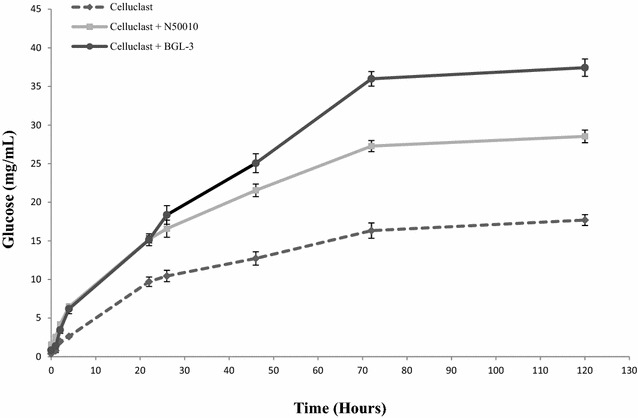
Glucose released from saccharification of wheat straw slurry. For the saccharification of 100 mg of wheat straw slurry, 1 U of BGL activity from Celluclast (the basic enzymatic cocktail) was supplemented with 1 U of either BGL activity from NS50010 (commercial BGL), or BGL-3 from *T. amestolkiae.* Glucose was determined in the samples after saccharification for 5 days (50 °C, 1200 rpm). All reactions were performed in triplicate

Efficient conversion of polysaccharides into glucose prior to yeast fermentation is an essential step for ethanol production, and BGLs are the key enzymes to achieve full depolymerization. The effectiveness of BGL-3* in the 2G process was evaluated following the saccharification of wheat straw slurry, one of the main lignocellulosic feedstocks used for production of bioethanol. The glucose released from the substrate using only Celluclast 1.5L (model source of cellulases with low β-glucosidase activity) was compared with the values determined for the same cocktail supplemented either with BGL-3* or NS-50010, a commercial cocktail rich in β-glucosidase. The results reflected the synergistic action of both BGL preparations with the cellulose cocktail (Fig. 6), but supplementation of Celluclast 1.5L with BGL-3 increased the degradation yield around 37% while with NS-50010 the increment was of only 17%. In view of these data, and remembering that NS-50010 is commercially sold as a β-glucosidase-rich preparation, the potential relevance of BGL-3* from *T. amestolkiae* for saccharification of lignocellulosic biomass must be emphasized.

### BGL-3 for laminarin saccharification

**Fig. 7:**
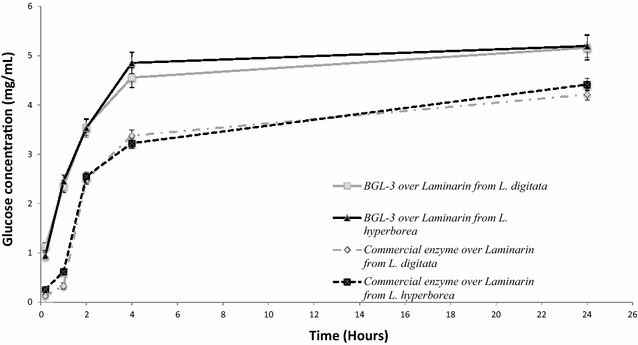
Glucose released from saccharification of laminarin from *L. digitata* and *L. hyperborea* using BGL-3 from *T. amestolkiae* and a commercial laminarinase. 100 mg of laminarin was incubated for 24 h with 3 U of laminarinase activity, at 50 °C, and 1200 rpm. All reactions were performed in triplicate

The versatility of the native and recombinant forms of the BGL-3 from *T. amestolkiae* prompted us to test the activity of the recombinant enzyme on hydrolysis of laminarin from two *Laminaria* species: *L. digitata* and *L. hyperborea.* Its efficiency on these substrates was compared with that of a commercial laminarinase (β-1,3 glucanase from *Helix pomatia*). Both the purified BGL-3* and the commercial β-1,3 glucanase hydrolyzed efficiently both laminarin samples in less than 24 h (Fig. 7), but BGL-3* worked better than the commercial enzyme at the same dose (3 U/mL). It should be noted that although the total BGL activity added to the reactions was identical, the dry weight of laminarinase necessary to get this activity was tenfold higher (0.01 mg/mL of BGL-3* vs. 0.1 mg/mL of β-glucanase from *Helix pomatia*). These data showed unequivocally that BGL-3* is more efficient for laminarin hydrolysis than an enzyme whose activity is specific for this substrate.

The versatility of some β-glucosidases to hydrolyze short oligosaccharides with β-1,2, β-1,3, β-1,4, or β-1,6 linkages has been widely reported [8–11]. However, the ability of these enzymes to degrade a polysaccharide like laminarin is much more exceptional and in spite of the higher affinity of the BGL-3 from *T. amestolkiae* against β-1,4 oligosaccharides, this catalyst releases a considerable amount of glucose from laminarin. Its efficiency has been compared with those of endo- and exo-β-1,3-glucanases. The results gathered in Table 4 show that the catalytic efficacy of BGL-3 on the laminarin samples tested was in the range of those reported for many enzymes that use laminarin as their natural substrate.

**Table 4:** Comparison of the kinetic constants of BGL-3 with other laminarin-degradative enzymes, using laminarin as substrate

Organism	Enzyme	*K*_m_	*k*_cat_	*k*_cat_ */K*_m_	References
*Podospora anserina*	PaGluc131A	4.1^a^	51.9^a^	12.6	[35]
*Pseudoalteromonas* sp. *Strain BB1*	ExoP	0.7	204.9	290.9	[36]
*Talaromyces emersonii*	Exo-1,3-β-glucanase	1.6	29.5	17.8	[37]
Paddy soil microbial metagenome	Umcel9y-1	47.3	127.5	2.6	[38]
*Aspergillus fumigatus*	1,3-β-glucanase	0.2	56.9^a^	219.1^a^	[30]
*Vibrio campbellii*	LamN	4.0	0.8	0.1	[33]
Barley -d-glucan	ExoII	0.1	28.0	230.0	[39]
	ExoI	0.1	73.0	740.0	[39]
*T. amestolkiae* BGL-3	BGL-3^b^	1.2	253.9	211.5	This work
	BGL-3^c^	1.1	247.9	225.3	This work

^a^ Calculated from data provided in the original article

^b^ Substrate: laminarin from *L. digitata*

^c^ Substrate: laminarin from *L. hyperborea*

The unexpectedly high activity of BGL-3 on a β-1,3 polysaccharide, together with its production in the absence of a carbon source, suggests its possible physiological role in *T. amestolkiae* cell wall metabolism. β-1,3-glucans are the major components of fungal cell walls of ascomycetes and basidiomycetes [23, 24]. Thus, a logical explanation can be that the fungus secretes BGL-3 for its autolysis, trying to find an alternative carbon source upon starvation from its own cell walls. This hypothesis is similar to that developed by Igarashi et al. [25], who reported an extracellular β-1,3 glucosidase from *Phanerochaete chrysosporium* and proposed its relationship with fungal cell wall metabolism. This process of autophagy was presented as a mechanism necessary to obtain glucose from the β-glucans present in its cell wall, sustaining fungal metabolism in the absence of an external carbon source.

## Conclusions

In this study, a very efficient β-glucosidase has been purified from *T. amestolkiae* cultures in a basal medium with glucose as carbon source, and characterized. This enzyme did not require cellulosic substrates to be produced, and was secreted in high amounts under carbon starvation. BGL-3 is a versatile enzyme able to hydrolyze β-1,4 oligosaccharides and β-1,3-glucans. Due to its outstanding properties, the enzyme was produced in *P. pastoris*. The purification yield of the recombinant protein was 7.5-fold higher than that of the native BGL-3 from *T. amestolkiae*, and both enzymes showed similar kinetic and physicochemical properties. This enzyme hydrolyzes efficiently lignocellulosic substrates and laminarin, and this catalytic versatility could be of great interest for depolymerization of different glucans for 2G and 3G bioethanol production. In summary, its easy production and purification, high efficiency, and versatility make BGL-3 an interesting candidate for biorefinery processes.

## Methods

### Microorganism and culture media

The ascomycete *T. amestolkiae* is deposited in the Collection of the Institute Jaime Ferrán of Microbiology (IJFM) at the Centro de Investigaciones Biológicas, with the access number A795. Fungal strains were maintained in tubes with PDA (Potato dextrose agar) medium, stored at 4 °C, and periodically reseeded in PDA plates, incubated at 28 °C.

To obtain spore suspensions from this culture, agar pieces (1 cm^2^) were cut and added to a tube containing 5 mL of a solution of 1% NaCl and 0.1% Tween 80. The mixture was used to inoculate 250 mL flasks with 50 mL of CSS medium (40 g/L glucose, 0.4 g/L FeSO_4_·7H_2_O, 9 g/L (NH_4_)_2_SO_4_, 4 g/L K_2_HPO_4_, 26.3 g/L corn steep solid, 7 g/L CaCO_3_, and 2.8 ml/L soybean oil), incubating at 28 °C and 250 rpm for 5 days. These cultures were used as pre-inoculum.

### Production and purification of BGL-3

For BGL-3 production, 2 mL from the CSS cultures of *T. amestolkiae* was inoculated in 250-mL Erlenmeyer flasks containing 50 mL of Mandels medium [26] with 1% of glucose as carbon source, and incubated in an Innova 4330 orbital shaker (New Brunswick Scientifics) at 28 °C and 250 rpm. All culture media were prepared with autoclaved distilled water.

When maximal β-glucosidase activity was detected in the supernatants (7 days), the cultures were cropped and centrifuged at 10,000×*g* for 30 min to separate mycelium and supernatant, which was vacuum-filtered through filter paper and nitrocellulose membrane discs (Millipore) of 0.8, 0.45, and 0.22 µm to complete clarification. This treated supernatant was further concentrated using a tangential flow filtration system (7518-02 Masterflex, from Millipore) equipped with a 10 kDa polysulfone membrane (Membrane Cassette, Filtron) and an ultrafiltration cell (Amicon, Millipore) with a 10 kDa cutoff polysulfone membrane (Millipore). Protein purifications were performed using an ÄKTA Purifier HPLC system (GE Healthcare Life Sciences). The crude extract was first dialyzed in 10 mM sodium phosphate, pH 6.0, and applied onto a Capto Adhere HiTrap cartridge (GE Healthcare Life Sciences) equilibrated with the same buffer at 2 mL/min. Retained proteins were eluted by using a linear gradient of NaCl in the same buffer (0–0.25 M of NaCl in 30 min) and 100% NaCl (15 min). Finally, the cartridge was equilibrated with the initial buffer.

The purified BGL-3 was dialyzed against acetate buffer, pH 4.0, and its homogeneity confirmed by SDS-PAGE using 10% gels stained with Coomassie brilliant blue R-250.

### *bgl*-*3* gene sequencing and real-time quantitative qRT-PCR analysis

In order to identify *bgl*-*3* sequence, a BLASTP against predicted proteins of *T. amestolkiae* was carried out. The gene sequences of the best hits were used as queries to run a local BLASTN against the assembled genome. An alignment between the gene and the best hits of the BLAST search was done to identify possible introns in the sequences of other BGLs. With the predicted coding sequence, the presence of a possible signal peptide was analyzed with the SignalP server, and the putative mature gene of BGL-3, without introns and signal peptide, was translated to protein by using the ExPASy Bioinformatics resource portal (ProtParam tool), in order to obtain the theoretical molecular mass and isoelectric point of BGL-3.

Primers for qRT-PCR were designed based on *bgl*-*3* sequence (BGL-3FWQPCR (TTCGTATCATGTCTGCATTC) and BGL-3RVQPCR (ATTCTTGAGGAGAACAATGC)). 18S rRNA was chosen for normalization of expression across all treatments [27] (primers 18sFW (ATTGGAGGGCAAGTCTGGTG) and 18sRV (CCAGTGAAGGCCATGGGATT)).

RNA was extracted from *T. amestolkiae* cultures growing in 1% of glucose using TRIzol reagent [28]. One-step qRT-PCR was performed using total RNA preparations treated with a Turbo DNA-free kit (Ambion). Brilliant III Ultra-Fast SYBR^®^ Green qRT-PCR Master Mix, from Agilent, was used for qRT-PCR reactions. Each reaction was performed according to the manufacturer’s instructions, adding 5 ng of the respective RNA.

Reactions were done in a LightCycler^®^ 96 detection system, and analyzed with LightCycler^®^ 96 SW. The running method consisted of several steps: 50 °C for 10 min, 95 °C for 3 min, 40 cycles of 95 °C for 10 s, and 60 °C for 20 s. All reactions were performed six times. The amplification efficiency for each primer pair was determined with serial dilutions from an RNA sample (100 ng RNA/µL) with at least five dilution points. The relative quantification of PCR products was calculated by the comparative 2^−ΔΔCT^ (cycle threshold) method.

### Cloning and expression of *bgl*-*3* in *P. pastoris*

RNA was isolated from fungal cultures by using TRIzol reagent, as explained before. The isolated transcripts were converted to cDNA using the Superscript II Reverse Transcriptase RT-PCR kit (Invitrogen) using 50 µM random hexamers. PCR amplifications were performed in a thermocycler Mastercycler pro S (Eppendorf) using genomic DNA as template. Primers were designed based on the nucleotide sequence of the *bgl3* gene from *T. amestolkiae* genome (GenBank accession no. MIKG00000000), but excluding the region corresponding to the signal peptide. Restriction sites for *XhoI* and *NotI* were, respectively, added to the forward and reverse primers (BG3FWXHOI: 5′-ATCTCGAGAAAAGATACTCTCCTCCAGCTTACCCT-3′, and BG3 RV NOTI: 5′-ATGCGGCCGCATGCCCAATCTTCAAAGCCAA-3′). Reaction mixtures were initially subjected to denaturation at 95 °C for 5 min, followed by 36 cycles of amplification consisting of denaturation at 95 °C for 45 s, primer annealing at 55 °C for 45 s, and elongation at 72 °C for 3 min, followed by a final extension step at 72 °C for 10 min. The PCR product was ligated to the yeast expression vector pPICzα (Invitrogen), and it was used for transforming *P. pastoris* X-33 after linearization with SacI (New England Biolabs). Transformed colonies were grown on YPD medium plates (10 g/L Yeast extract, 20 g/L peptone, 20 g/L glucose, and 10 g/L of agar) with 100 μg/mL of zeocin as selection marker. Since it is considered that the better the zeocin resistance, the better the protein production, the scored transformants were re-screened for resistance to a zeocin concentration of 1 mg/mL, selecting the clones with the highest tolerance for protein production.

### Production and purification of recombinant BGL-3

To prepare a fresh inoculum, the selected clones were grown overnight in 250 mL flasks with 50 mL of YEPS medium at 28 °C and 250 rpm. Then, recombinant protein production was carried out in 2-L flasks with 400 mL of YEPS medium (20 g/L peptone, 10 g/L yeast extract, 10 g/L sorbitol). Cultures were incubated at 28 °C and 250 rpm for 7 days with daily addition of 5 g/L methanol. Samples were periodically taken to measure β-glucosidase activity.

For BGL-3* purification, 7-day-old cultures were harvested and centrifuged at 10,000×*g* and 4 °C for 20 min. The supernatant was first concentrated by tangential filtration and finally concentrated and dialyzed against 10 mM phosphate buffer (pH 6.0) using a 50-kDa cutoff membrane (Merck-Millipore). BGL-3* was purified after two chromatographic steps. First, a QFF Hi Trap cartridge (GE Healthcare) equilibrated with phosphate buffer pH 6.0 was used. Elution of the bound proteins was carried out by applying a linear gradient from 0 to 0.25 M of NaCl in 25 min, at 2 mL/min. The column was then washed with 10 mL of 1 M NaCl and re-equilibrated using 10 mL of the starting buffer. Fractions with β-glucosidase activity were collected, dialyzed, and concentrated. To complete the purification of BGL-3*, the sample from the previous stage was analyzed by size exclusion chromatography on Superose 12 column (GE Healthcare Life Sciences). To avoid unspecific interactions, the same buffer (plus 100 mM NaCl) was used for column equilibration and proteins elution, at a flow of 0.5 mL/min.

### Protein quantification, enzyme assays, and substrate specificity

Total protein was estimated by the BCA method using bovine serum albumin as standard, measuring the absorbance of the sample at 280 nm in a Nanodrop (Thermo Fisher Scientific). The β-glucosidase standard reaction was performed using 0.1% (w/v) *p*-nitrophenyl-β-d-glucopyranoside (*p*NPG, Sigma), at 70 °C, in sodium acetate buffer 100 mM, pH 4.0. Other nitrophenyl derivatives, as *p*NPX (*p*-nitrophenyl-β-d-xylopyranoside), β-*p*NPgal (*p*-nitrophenyl-β-d-galactopyranoside), α-*p*NPG, α-*p*NPgal (*p*-nitrophenyl-α-d-galactopyranoside), *p*-nitrophenyl-α-L-rhamnopyranoside, and *p*-nitrophenyl-β-d-fucopyranoside were assayed analyzing *p*NP release. The reactions were stopped after 10 min by adding 2% (w/v) Na_2_CO_3_, and the *p*NP released was spectrophotometrically measured at 410 nm. One BGL activity unit was defined as the amount of enzyme capable of releasing 1 micromole of *p*NP per minute (the molar extinction coefficient of *p*NP is 15,200 M^−1^ cm^−1^).

β-glucosidase activity on cellobiose, gentiobiose, laminaribiose, cellotriose, cellotetraose, cellopentaose, and cellohexaose, maltose, sucrose, and lactose, was quantified by measuring the glucose released from these compounds after enzyme hydrolysis, using the Glucose-TR commercial kit (Spinreact), according to the manufacturer’s instructions. Reactions were performed in sodium acetate 100 mM, pH 4.0, incubating in a heating block for 10 min at 1200 rpm. Then, the reactions were stopped by heating at 100 °C for 5 min.

The activity of BGL-3 was also determined against different polysaccharides, all of them prepared in 50 mM sodium acetate buffer, pH 4.0: 1.25% Avicel (microcrystalline cellulose), 3% carboxymethyl cellulose (CMC), 1% laminarin from *L. digitata* and *L. hyperborea*, and 3% beechwood xylan. The substrates were incubated with BGL-3 in a heating block at 60 °C and 1200 rpm for 10 min. The released reducing sugars were determined by the Somogyi–Nelson method [29]. A degree of polymerization of 25 units has been previously described for laminarin [30].

The kinetic constants of the purified BGL-3 were determined against *p*NPG (over a range of concentrations from 10 μM to 5 mM), *o*NPG (40 μM to 20 mM), cellobiose (80 μM to 40 mM), gentiobiose (80 μM to 40 mM), laminaribiose (80 μM to 40 mM), cellotriose (80 μM to 40 mM), cellotetraose (80 μM to 40 mM), cellopentaose (40 μM to 20 mM), and cellohexaose (20 μM to 10 mM). The values of *K*_m_ and *V*_max_ were determined using the program SigmaPlot, based on the Michaelis–Menten model.

*K*_i_ for BGL-3 was calculated using *p*NPG as substrate, in the presence of different concentrations of glucose (0, 2.5, 5, and 10 mM).

All enzymatic assays were performed including 0.1% BSA, a protein which does not affect the catalytic activity of the BGL-3, to prevent the activity loss when working with low enzyme concentrations [15].

### Physicochemical properties

To obtain the peptide mass fingerprint of the protein, the sample was run in a SDS-PAGE gel as explained before, excising the BGL-3 band. After tryptic digestion [31], the peptides’ mixture was analyzed in a MALDI-TOF/TOF Autoflex III (Bruker Daltonics) equipped with a laser and a Smartbeam LIFT-MS/MS device. The data from MS and MS/MS experiments were combined using the 3.0 BioTools (Bruker Daltonics) software and searched against the NCBInr database using 2.3 MASCOT as the search engine (Matrix Science). Relevant search parameters were trypsin as enzyme, carbamidomethylation of cysteines as fixed modification, methionine oxidation as variable modification, 1 missed cleavage allowed, peptide tolerance of 50 ppm, and MS/MS tolerance of 0.5 Da. Protein scores greater than 75 were considered significant.

The molecular mass of the native BGL-3 was determined both by size exclusion chromatography using a Superose 12 column (GE Healthcare), and by MALDI-TOF in the instrument described above. Isoelectric point (pI) was determined by isoelectrofocusing (IEF) in 5% (w/v) polyacrylamide gels, prepared with Pharmalyte (pH 3.0–10.0) as carrier ampholytes (GE Healthcare), using a Mini Protean III Cell system (Bio-Rad). 1 M H_3_PO_4_ and 1 M NaOH were the anode and cathode buffers, respectively. The pH gradient was directly measured on the gel using a contact electrode (Crison). The activity of BGL-3 was tested in zymograms after IEF, incubating the gel with 2 mM *p*-methylumbelliferyl-β-d-glucopyranoside (Sigma-Aldrich) for 10 min, and observing the gel under UV light with a Gel Doc XR + system (Bio-Rad) to detect free 4-methylumbelliferone.

The optimal values of pH and temperature and the stability of BGL-3 were evaluated with *p*NPG as model substrate, measuring the residual activity after the treatments in standard conditions. The buffer Britton–Robinson (100 mM) was used to study the effect of pH on BGL-3 activity, adjusting different aliquots to pH values from 2.0 to 10.0. BGL-3 was incubated at 4 °C and different pH values for 3 days. After this time, a standard BGL reaction was performed to determine its optimal pH. Temperature assays were done between 30 and 80 °C using solutions of BGL-3 in acetate pH 4.0. Its thermostability was analyzed in the same temperature range for 72 h, taking aliquots at different incubation times to measure the residual activity.

### Saccharification of wheat straw slurry and laminarin

Enzymatic saccharification was tested in samples of wheat straw slurry from steam explosion (kindly provided by Abengoa). For saccharification, 100 mg of wheat straw slurry was treated with 2 U/mL of BGL activity in 100 mM sodium acetate buffer, pH 4 (final volume of 2 mL), incubating in a heat block at 50 °C and 1200 rpm, for 120 h. The sources of BGL activity tested were Celluclast 1.5L (Novozymes), a basal cocktail for biomass degradation with low BGL activity, NS-50010 (Novozymes), which is a β-glucosidase-rich cocktail, and the purified BGL-3. The control sample contained Celluclast 1.5L as the unique source of BGL activity. To compare the efficiencies of NS-50010 and BGL-3, 1 U/mL of BGL activity from Celluclast 1.5L was supplemented with 1 U/mL of either NS-50010 or the purified BGL-3. The glucose released was measured in sample supernatants at different time intervals, using the Glucose-TR commercial kit (Spinreact), according to the manufacturer’s instructions.

Similarly, the release of glucose from laminarin, from *Laminaria digitata* (Sigma-Aldrich) and *Laminaria hyperborea* (Koch-light laboratories), was evaluated. The reaction mixtures contained 100 mg of laminarin in 10 mL of 100 mM sodium acetate buffer, pH 4.0 and 3 U/mL of laminarinase activity. This was provided by either the purified BGL-3 or a commercial β-1,3-glucanase from *Helix pomatia* (Sigma-Aldrich). Reactions were performed in a heat block at 50 °C and 1200 rpm for 24 h, measuring the glucose released at different times as detailed above.

## Additional file

**Additional file 1: Table S1.** Specific activity of BGL-3 against other glycosides, disaccharides and polysaccharides. **Table S2.** Purification of recombinant BGL-3. **Figure S1.** DNA sequence of *bgl3*. The predicted signal peptide is underlined. Predicted introns are indicated in red. **Figure S2.** Structural comparison between BGL-3 (cyan) and a barley β-D-glucan glucohydrolase isoenzyme in complex with 4’-nitrophenyl 3I-thiolaminaritrioside (SMTL ID 1j8v.1, orange). The BGL-3 model was generated using the SWISS-MODEL server, based on sequence similarity, using a β-glucosidase from *Aspergillus aculeatus* as template (SMTL ID 4iib.1). Q Mean, coverage and sequence identity for this model were − 0.47, 0.96 and 65.75% respectively. The PyMol v0.99 program was used to visualize, analyze and align the structures. Active site residues D254 and E484 on BGL-3 are marked as cyan sticks, residues implied in substrate binding on glucohydrolase appear highlighted as orange sticks. **Figure S3.** Relation between fungal growth (in Mandels medium with glucose as carbon source) and β-glucosidase activity, during the first 24 h of culture.
