# Supplementary material for: The β-glucosidase secreted by Talaromyces amestolkiae under carbon starvation: a versatile catalyst for biofuel production from plant and algal biomass
Source: Biotechnol Biofuels. 2018 Apr 27;11:123. doi: 10.1186/s13068-018-1125-9 (PMC5921417; doi:10.1186/s13068-018-1125-9)

Table S1. Specific activity of BGL-3 against other glycosides, disaccharides and polysaccharides.

Substrate	Specific activity (U/mg)
***p*NPX (*p*-nitrophenyl-β-D-xilopyranoside)**	7.58±0.24
**β-*p*NPgal (*p*-nitrophenyl-β-D-galactopyranoside)**	0.06±0.01
**α-*p*NPG (*p*-nitrophenyl-α-D-glucopyranoside)**	6.26±0.12
**α-pNPgal (*p*-nitrophenyl-α-D-galactopyranoside)**	0
***p*-nitrophenyl-α-L-rhamnopyranoside**	0
***p*-nitrophenyl-β-D-fucopyranoside**	0
**Maltose**	3.42±0.14
**Sacarose**	0.26±0.01
**Lactose**	0.26±0.12
**1.25% Avicel**	7.01±0.27
**3% Xylan**	12.61±0.39
**3% Carboxymethyl cellulose (CMC)**	10.56±0.21

Table S2. Purification of recombinant BGL-3

**BGL-3* Purification**				
Step	Total protein (mg)	Total activity (U)	Specific activity (U/mg)	Yield (%)
Crude extracts	22.17	287.36	19.96	100
HiTrap QFF	2.83	156.43	55.27	54.43
Superose 12	0.21	43.63	207.78	54.27

Figure S1. DNA sequence of *bgl3*. The predicted signal peptide is underlined. Predicted introns are indicated in red.

ATGCGGAACAGTTTATTGATTTCGCTTGCTGCGGCAGCACTTGCCGAGGGCAAGGCC**TAC**TCTCCTCCAGCTTACCCT
GCTCCCTGGGCCAGTGGCGCCGGGGAATGGGCTCAAGCTCATGACAGAGCAGTCGAGTTCGTTTCGCAATTGACCTTG
GCCGAGAAGATAAACCTGACGACTGGTGTTGG**GTACGTTGTGATACCGTTGAGCAAAATAAAGACATGGGACTGACAA**
TTGGTAGATGGGAGGGTGGACAATGTGTCGGTAACACTGGAAGCATTCCCCGCCTGGGATTCCGCAGCCTCTGTATGC
AGGATTCACCGCTCGGCGTGAGAGACA**GTATGTCGTGCCCTGTAGTCTCTTGCCTCTTACTTCTTACCGTCCAAATAC**
AAAAAGAGAAAAAAAAAAAAGGTTGAATTATATAGCTAACAGTTTTATGTTTCTAGCTGACTACAATACTGCCTTCCCT
GCTGGCGTCAATGTCGCCGCTACCTGGGATCTCGATCTTGCATACCGGCGCGGTGTAGCCATGGCTGAGGAACACCGT
GGCAAAGGTGTGGATGTTCAGCTTGGACCCGTTGCTGGTCCGCTAGGAAGAGCACCAGAGGGTGGCCGTAATTGGGAA
GGCTTTGCACCCGACCCCGTTTTGACTGGTCAGATGATGGCAAGCACTATTGAAGGAATGCAGGATACCGGTGTGATT
GCTTGTGCAAAGCACTATATCGGTAATGAGCAAGAGCACTTTCGTCAGGGCTCCCAGGAAAATTATACAGTCGCTGAT
GCTATCAGCTCGAACATCGACGATGTTACTTTGCACGAGTTGTACCTGTGGCCGTTTGCCGATGCGGTTAGGGCAGGT
GTTGGTTCCGTCATGTGTTCTTACAATCAATTGAACAACAGTTATTCCTGCGGCAACAGCTACAGTTTGAACCACATT
CTTAAGGGAGAACTCGACTTTCAAGGATTCGTCATGACCGACTGGGGTGCTCAGCATTCTGGTGTGGGCGATGCTTTG
GCCGGTGCGGATATGGATATGCCTGGTGATGTGGCTTTCGACAGTGGAACTGCTTTCTGGGGTACTAACTTGACAATT
GCCGTGCTCAATGGAACTGTTCCTGAATGGCGTATTGACGACATGGCCGTTCGTATCATGTCTGCATTCTATAAGGTT
GGTCGTGATCGTACCCAGGTCCCCATCAACTTTGCTAGCTGGACTCTGGATACCTATGGCAATGAATACTACTACGCC
GGCGAGGGCTACAAGGAAATCAACCAGCACGTTGATGTACGTGGTGACCACGCCAAAGTTGTCCGTGAAATCGGCAGT
GCCAGCATTGTTCTCCTCAAGAATGTTGACGGCGCTCTTCCGTTGACTGGCTCCGAGAGGTTTGTCGCAGTTTTCGGA
GAGGATGCTGGCTCCAATCCTGATGGTGTCAATGGTTGCTCTGACCGTAACTGTGATAACGGTACCTTGGCTATGGGA
TGGGGTAGTGGTACTGCCAACTTCCCTTACCTAGTTACTCCTGAACAAGCTATCCAAGCCGAGGTCTTGAAGAATGGC
GGAATATTTACTGCTATTACCGACAGCGGCGCCACCAATACTACAGCCACGACCGTGGCTGCTCAAGCCTC**GTAAGTA**
CTGGTCGTAAGTAGAACATAGCAATGGCTCAGATTACTGACTTTGTTTTAATTAGGGCTTGCCTAGTGTTTGCCAATG
CAGACTCCGGCGAGGGATACATCACCGTTGACGGAAACGTGGGAGATCGTAAGAATTTGACATTATGGCAGAACAGTG
AAGCTATGATCTCGGCCGTTGCAGGTAACTGTAACAACACCATAGTAGTTCTTCATACTGTTGGACCTGTTCTCGTTG
AGGACTGGGTCAACCATCCCAACATCACTGCTGTTTTGTGGGCAGGTTTGCCTGGAGAGCAGAGCGGAAACTCTTTGG
TTGATGTTCTTTACGGCAGCGTCAACCCCGGAGGCAAGACTCCTTTCACTTGGGGCAAGCAACGTTCTGACTGGGGAA
CCGATATCATCTACGAACCCAACAACGGAGATGGTGCTCCTCAGCAGGACTTCACCGAGGGTATCTTCATTGACTACC
GACACTTTGATAAATACAATATTACTCCCACTTACGAGTTTGGTTATGGTCTCAGTTACAGCACCTTCTCTTTCTCAA
ATCTCCAGGTGACTCCTCTCGCTGCTTCGCCTTACAAACCAGCCACAGGTCACAGCGGTCCCGCACCTGTCCTGGGCA
AGGTTTTGAACGCCACGGCTTATCTTTTCCCTAACTACATCAAACGCATTGAAGCTTTCATTTACCCATGGCTTAACT
CCACTGATCTGAGGACTTCCTCTGGTGATCCAAATTACGGTTGGTCTACTTCCAAATACGTGCCTGACGGTGCTCAAG
ACGGATCTCCGCAACCTGTCAACCCCGCTGGTGGTGCCCCTGGTGGCAACCCTGCGCTGTATGACCCTGTTGCCGAAA
TCAGAGTGACTGTCAAGAACACCGGAAAGGTCGCTGGTGTTGAAGTGCCTCAGCTCTATGTCTCGCTCGGTGGCCCCT
CCGATGCGCCTAAGGTTCTTCGTGGCTTTGGCCGCCTTTCTCTCGGTGCTGGCGAGGAGGCTCAGTGGACTGCCACTT
TGACCCGACGTGACGTTTCTAATTGGGACACTGTCAGCCAGAACTGGGTTGTCTCAAACTACACCAAGACTGTCTATG
TCGGCAACTCTTCTCGCAACTTGCCGCTCCAGCAGACTTTGGCTTTGAAGATTGGGCAT**TAA**

Figure S2: Structural comparison between BGL-3 (cyan) and a barley βD-glucan glucohydrolase isoenzyme in complex with 4'-nitrophenyl 3I-thiolaminaritrioside (SMTL ID 1j8v.1, orange). The BGL-3 model was generated using the SWISS-MODEL server, based on sequence similarity, using a βglucosidase from *Aspergillus aculeatus* as template (SMTL ID 4iib.1). Q Mean, coverage and sequence identity for this model were -0.47, 0.96 and 65.75% respectively. The PyMol v0.99 program was used to visualize, analyze and align the structures. Active site residues D254 and E484 on BGL-3 are marked as cyan sticks, residues implied in substrate binding on glucohydrolase appear highlighted as orange sticks.

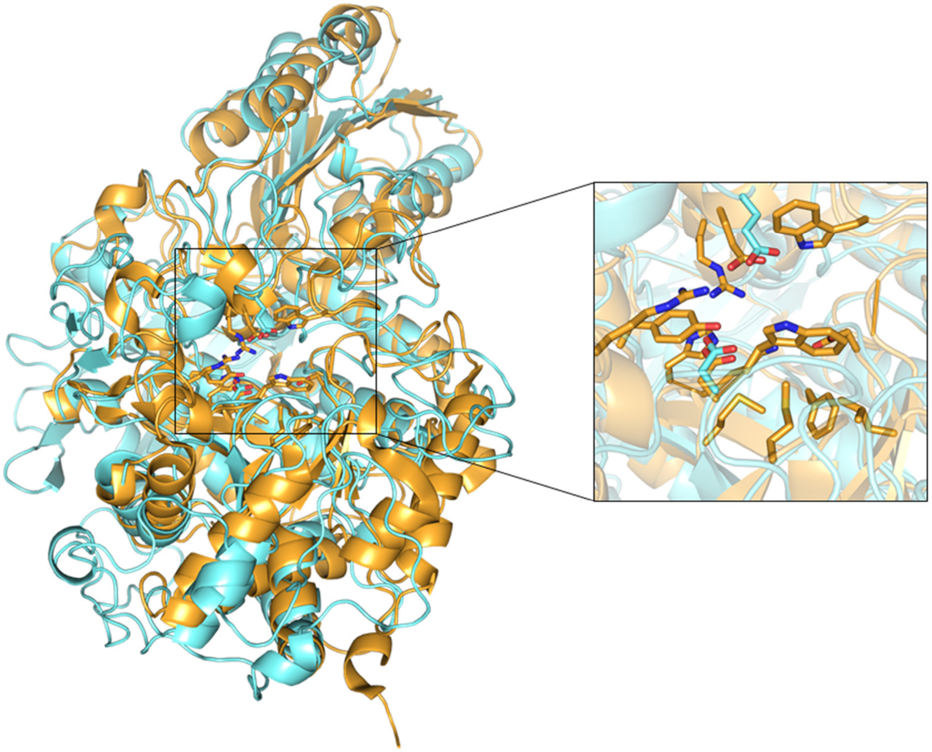

Figure S3. Relation between fungal growth (in Mandels medium with glucose as carbon source) and β-glucosidase activity, during the first 24 h of culture.

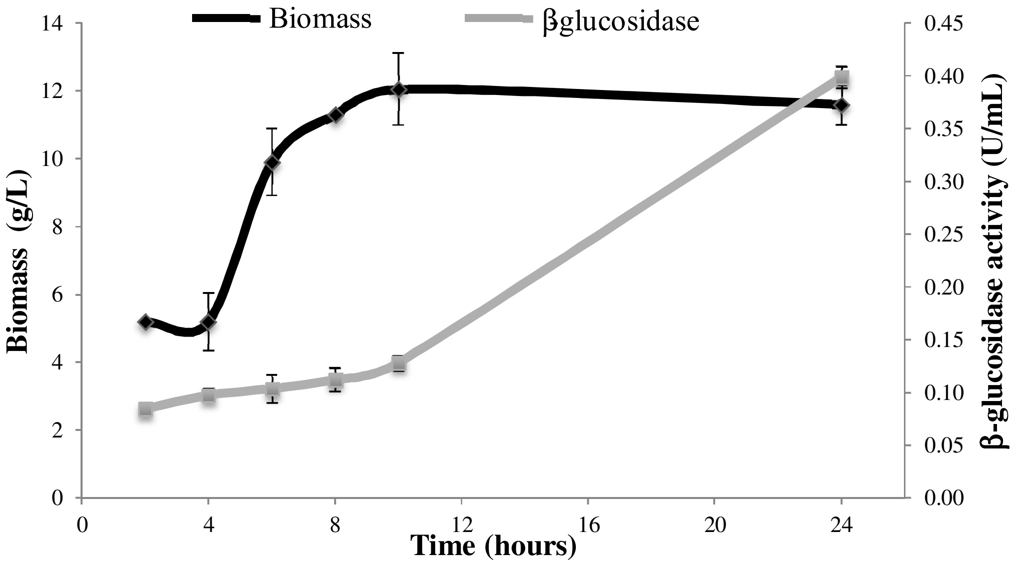

Supplement: Supplementary file 1 — Additional file 1: Table S1. Specific activity of BGL-3 against other glycosides, disaccharides and polysaccharides. Table S2. Purification of recombinant BGL-3. Figure S1. DNA sequence of bgl3. The predicted signal peptide is underlined. Predicted introns are indicated in red. Figure S2. Structural comparison between BGL-3 (cyan) and a barley β-D-glucan glucohydrolase isoenzyme in complex with 4’-nitrophenyl 3I-thiolaminaritrioside (SMTL ID 1j8v.1, orange). The BGL-3 model was generated using the SWISS-MODEL server, based on sequence similarity, using a β-glucosidase from Aspergillus aculeatus as template (SMTL ID 4iib.1). Q Mean, coverage and sequence identity for this model were − 0.47, 0.96 and 65.75% respectively. The PyMol v0.99 program was used to visualize, analyze and align the structures. Active site residues D254 and E484 on BGL-3 are marked as cyan sticks, residues implied in substrate binding on glucohydrolase appear highlighted as orange sticks. Figure S3. Relation between fungal growth (in Mandels medium with glucose as carbon source) and β-glucosidase activity, during the first 24 h of culture. [file 13068_2018_1125_MOESM1_ESM.pdf]
